# Identifying Areas of the Visual Field Important for Quality of Life in Patients with Glaucoma

**DOI:** 10.1371/journal.pone.0058695

**Published:** 2013-03-08

**Authors:** Hiroshi Murata, Hiroyo Hirasawa, Yuka Aoyama, Kenji Sugisaki, Makoto Araie, Chihiro Mayama, Makoto Aihara, Ryo Asaoka

**Affiliations:** 1 Department of Ophthalmology, University of Tokyo Graduate School of Medicine, Tokyo, Japan; 2 Tokyo Koseinenkin Hospital, Tokyo, Japan; 3 Kanto Central Hospital, The Mutual Aid Association of Public School Teachers, Tokyo, Japan; 4 Shirato Eye Clinic, Tokyo, Japan; Univeristy of Melbourne, Australia

## Abstract

**Purpose:**

The purpose of this study was to create a vision-related quality of life (VRQoL) prediction system to identify visual field (VF) test points associated with decreased VRQoL in patients with glaucoma.

**Method:**

VRQoL score was surveyed in 164 patients with glaucoma using the ‘Sumi questionnaire’. A binocular VF was created from monocular VFs by using the integrated VF (IVF) method. VRQoL score was predicted using the ‘Random Forest’ method, based on visual acuity (VA) of better and worse eyes (better-eye and worse-eye VA) and total deviation (TD) values from the IVF. For comparison, VRQoL scores were regressed (linear regression) against: (i) mean of TD (IVF MD); (ii) better-eye VA; (iii) worse-eye VA; and (iv) IVF MD and better- and worse-eye VAs. The rank of importance of IVF test points was identified using the Random Forest method.

**Results:**

The root mean of squared prediction error associated with the Random Forest method (0.30 to 1.97) was significantly smaller than those with linear regression models (0.34 to 3.38, p<0.05, ten-fold cross validation test). Worse-eye VA was the most important variable in all VRQoL tasks. In general, important VF test points were concentrated along the horizontal meridian. Particular areas of the IVF were important for different tasks: peripheral superior and inferior areas in the left hemifield for the ‘letters and sentences’ task, peripheral, mid-peripheral and para-central inferior regions for the ‘walking’ task, the peripheral superior region for the ‘going out’ task, and a broad scattered area across the IVF for the ‘dining’ task.

**Conclusion:**

The VRQoL prediction model with the Random Forest method enables clinicians to better understand patients’ VRQoL based on standard clinical measurements of VA and VF.

## Introduction

Vision-related quality of life (VRQoL) can be defined as a person’s satisfaction with their visual ability and how their vision impacts on their daily life [1]. In glaucoma patients, visual field (VF) loss [2]–[11] and reduced visual acuity (VA) [8]–[14] impact on VRQoL; however, these studies only investigated the influence of summary measures, such as mean deviation (MD), on VRQoL. Very few reports have attempted to identify the areas of the VF that are important for different daily tasks; Sumi et al. reported that retinal sensitivity in the lower hemifield within 5° of fixation, and better eye VA, play the most important role in VRQoL [15] while other studies have suggested the importance of other VF regions for specific tasks, such as driving [16] and postural stability [17]. Other reports have revealed that glaucomatous VF damage has an effect on hand-eye coordination [18], the likelihood of falling [19], the possibility of causing or being involved in a motor vehicle accident [19]–[23] (likely due to an inability to detect peripheral obstacles and hazards [16], [21]), and the risk of fractures [24].

The two most significant measures of visual function, VA and VF sensitivity, are correlated in glaucoma patients [7], especially when glaucomatous damage affects the central VF [25], [26]. Furthermore, VF sensitivities of neighboring test points are also correlated [27]–[29]; therefore, this spatial relationship should also be taken into account when analyzing the relationship between the VF and VRQoL. Nonetheless, most previous studies have investigated the impact of VA, and VF sensitivity in different regions of the VF, on VRQoL separately [9]–[15]. Indeed, previous studies have reported that the relationship between VRQoL and VF sensitivity attenuates when the relationship is adjusted for VA [8], [10]. In the current study, we have used Breiman’s ‘Random Forest’ machine learning algorithm [30] to predict VRQoL, and to identify the most important VF test points for a number of different daily tasks since this method can cope with highly correlated predictor variables. [31]. Indeed, the Random Forest algorithm has be used to explore interactions between different predictor variables[31]–[33]; thus, VA and VF sensitivity can be considered concurrently, and the spatial relationship between neighboring VF test points will not bias the results.

The purpose of this study is to generate a method to predict a glaucoma patient’s VRQoL based on their VA and VF sensitivity, considering their inter-correlation, and to identify the areas of the VF most important for different daily tasks.

## Materials and Methods

The study was approved by the Research Ethics Committee of the Graduate School of Medicine and Faculty of Medicine at the University of Tokyo. Written consent was given by the patients for their information to be stored in the hospital database and used for research. This study was performed according to the tenets of the Declaration of Helsinki.

The subjects of the current study included 164 patients (86 males and 78 females) with glaucoma (85 patients with primary open-angle glaucoma, 72 patients with normal tension glaucoma, 4 patients with primary angle-closure glaucoma, and 3 patients with secondary open angle glaucoma). All of the study patients were recruited at the glaucoma clinic in the Hospital of the University of Tokyo. All patients enrolled in the study fulfilled the following criteria: (1) glaucoma was the only disease causing VF damage and/or VA impairment; (2) patients were followed for at least 6 months at the University of Tokyo Hospital. The VF was evaluated using the Humphrey Field Analyzer (Carl Zeiss Meditec, Dublin, CA) 30-2 Swedish Interactive Threshold Algorithm (SITA) standard program with reliable results: fixation losses <25%, and false-positive error <15%; false negative rate was not used, as a reliability criterion because it is has been shown to be positively correlated with the level of VF damage rather than patient attentiveness. [34]. All of the patients had a glaucomatous VF defect in at least one eye defined as three or more contiguous total deviation points at p<0.05, or two or more contiguous points at p<0.01, or a 10 dB difference across the nasal horizontal midline at two or more adjacent points, or MD worse than −5 dB [1]. VF damage was stable with medically- or surgically-controlled intraocular pressure for at least 2 years.

Characteristics of the study sample are summarized in **Table 1**. The mean (± standard deviation) age of patients was 61.9±12.1 years, ranging from 26 to 89 years. The MD of the better eye was −13.1±9.3 (range: −31.4 to 1.96) dB, while the MD of the worse eye was −17.9±9.6 (range: −33.2 to 0.4) dB. Mean best corrected VA in the logarithm of the minimum angle of resolution (logMAR) was 0.09±0.43 (range: −0.30 to 2.6) in the better eye and 0.52±0.9 (range: −0.30 to 2.8) in the worse eye.

**Table 1:** Patient demographics.

Age, y, mean ± SD (range)	61.9±12.1 (26 to 89)
Gender (male : Female)	86∶78
MD of better eye, dB, mean ± SD (range)	−13.1±9.3 (−31.4 to 2.0)
MD of worse eye, dB, mean ± SD (range)	−17.9±9.6 (−33.2 to 0.4)
Visual acuity of better eye, mean ± SD (range)	0.09±0.43 (−0.30 to 2.6)
Visual acuity of worse eye, mean ± SD (range)	0.52±0.9 (−0.30 to 2.8)
Type of glaucoma (POAG, NTG, PACG, SOAG)	85, 72, 4, 3

MD: mean deviation, SD: standard deviation, POAG: primary open-angle glaucoma, NTG: normal tension glaucoma, PACG: primary angle-closure glaucoma, and SOAG: secondary open angle glaucoma.

VRQoLwas assessed using the method developed by Sumi et. al. [15]. Briefly, the ‘Sumi Questionnaire’, written in Japanese, contains 30 questions regarding 7 tasks: legibility of letters (‘letters’), legibility of sentences (‘sentences’), walking, using public transportation (‘going out’), dining, dressing, and additional miscellaneous activities (‘miscellaneous’) (see **Table 2**, note that questions have been translated into English for this article). The Sumi questionnaire also includes one question (question 7) regarding the difficulty in reading vertically, since this is the traditional way to read/write sentences in Japanese. Each question is associated with three possible responses, scored as follows; greatly disabled (2 points), slightly disabled (1 point), and not disabled (0 points). Mean score was calculated for each of the seven tasks to attain a visual disability index. The ‘letters’ and ‘sentences’ tasks were merged since these questions are closely related. The ‘dressing’ task was not analyzed because of the small number of questions (n  = 2), but its score was used in the calculation of overall VRQoL. Within 3 months of visual disability assessment, we tested the VF in both eyes with a 5-minute rest between each eye examination.

**Table 2:** Questions included in the ‘Sumi Questionnaire’ (questions originally written in Japanese).

**Legibility of letters: letters**
1. Can you read the headlines of a newspaper? (Yes/With difficulty/No)
2. Can you read small print in a newspaper? (Yes/With difficulty/No)
3. Can you read words in a dictionary? (Yes/With difficulty/No)
4. Can you see the numbers in a telephone directory? (Yes/With difficulty/No)
5. Can you make out a fare table for trains and subways? (Yes/With difficulty/No)
**Sentences**
6. Do you have difficulty reading and writing? (No/Occasionally/Frequently)
7. When you write sentences in vertical lines, does it lean to either direction? (No/Occasionally/Frequently)
8. When you read, can you find the next line easily? (Yes/With difficulty/No)
**Walking**
9. Do you have difficulty walking because of your visual problems? (No/Occasionally/Frequently)
10. Can you take a walk by yourself? (Yes/With difficulty/No)
11. Do you misjudge traffic signals? (No/Occassionally/Frequently)
12. Do you bump into people or objects while walking? (No/Occasionally/Frequently)
13. Do you stumble on the stairs? (No/Occasionally/Frequently)
14. Do you fail to notice changes in the ground? (No/Occasionally/Frequently)
15. Do you fail to recognize your friends until they talk to you? (No/Occasionally/Frequently)
16. Do you fail to see people or cars approaching you from the side? (No/Occasionally/Frequently)
**Going out**
17. Do you have difficulty going out because of your visual problems? (No/Occasionally/Frequently)
18. Do you need somebody to accompany you to go to new places? (No/Preferably/Yes)
19. Can you get a cab by yourself? (Yes/With difficulty/No)
20. Do you have difficulty traveling by train? (No/Occasionally/Frequently)
21. Do you feel uneasy going out at night because of your visual problems? (No/Occasionally/Frequently)
**Dining**
22. Do you have difficulty dining because of your visual problems? (No/Occasionally/Frequently)
23. Do you drop food while dining because of your visual problems? (No/Occasionally/Frequently)
24. Do you spill tea while pouring into a cup? (No/Occasionally/Frequently)
25. Do you have difficulty using chopsticks? (No/Occasionally/Frequently)
**Dressing**
26. Do you ever button up clothing in the wrong order? (No/Occasionally/Frequently)
27. Can you see your face clearly in the mirror? (Yes/With difficulty/No)
**Miscellaneous**
28. Can you recognize people’s faces on TV? (Yes/With difficulty/No)
29. Do you have difficulty finding objects dropped on the floor? (No/Occasionally/Frequently)
30. Do you have difficulty dialing the telephone? (No/Occasionally/Frequently)

**Figure 1:**
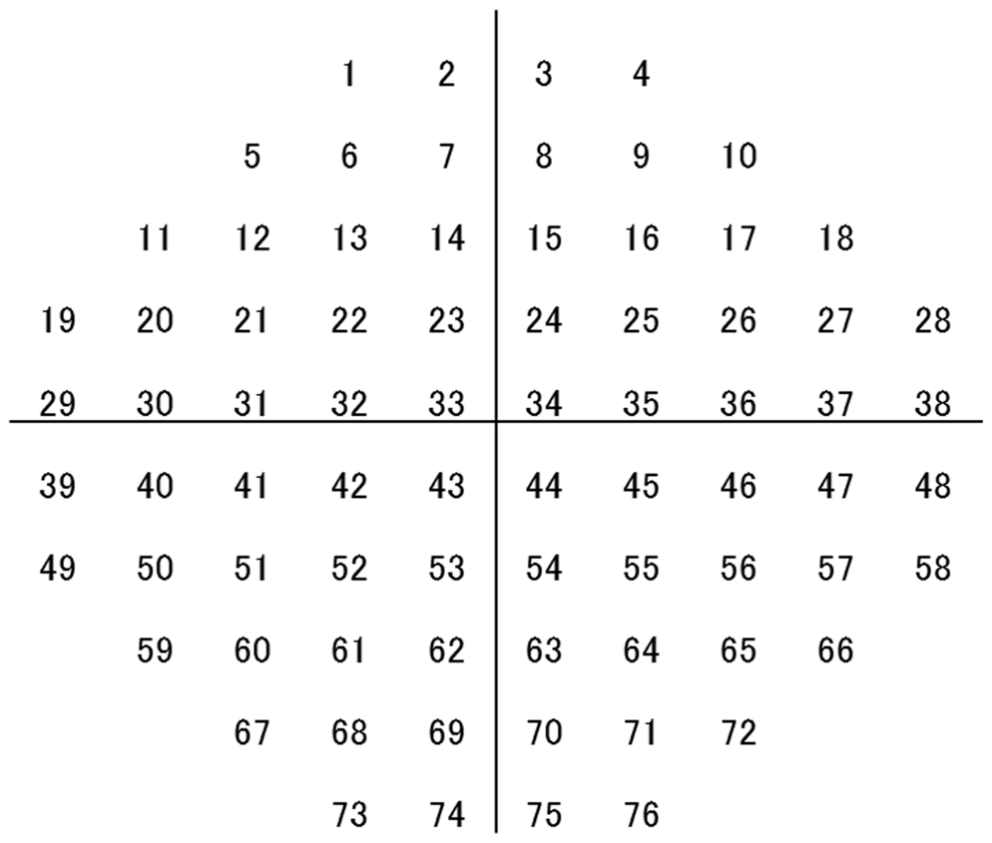
Integrated visual field test point locations.

To analyze the relationship between VF sensitivity at each test point and VRQoL, a binocular VF was calculated for each patient by merging a patient’s monocular HFA VFs using the “best sensitivity” method (integrated VF (IVF)) [35]–[38]. In short, each point on a monocular VF has a spatially corresponding point on the VF of the fellow eye in binocular viewing. In the IVF, the sensitivity and TD at each point was calculated using the maximum raw sensitivity (dB) and best TD (least negative) value from each of the two overlapping points, as if the subject was viewing binocularly (**Figure 1** illustrates the numeric locations of all IVF test points).

The better- and worse-eyes were determined using the MD values (better-eye had less damaged MD). For the analysis of VA, logMAR was used.

The relationships between TD values, better-eye VA, worse-eye VA, TD values and age, and VRQoL scores were analyzed using the Random Forest method. The Random Forest algorithm is an ensemble classifier proposed by Breiman in 2001 [30], which consists of many decision trees and outputs the averaged value of all the individual trees. Each tree is constructed using a different bootstrap sample from the original data (bootstrapping is repeated until the sample size reaches the original sample size, allowing duplication). Thus, cross-validation is performed internally, removing the need for a separate cross-validation dataset to obtain an unbiased estimate of the test set error. Next, the optimal regression model is determined by a measure of model fitness to the data (mean-square error for linear regression). A particular merit of the Random Forest method is that any interaction or correlation between predictor variables can be taken into account. Subsequent partitioning of the data with a different predictor from the prior partition in the decision tree, which is the weak learner of the Random Forest method, represents an interaction effect, and consequently the decision tree can represent high order interactions. In addition, each predictor has less opportunity to compete against correlated predictors since each predictor is selected randomly for each stage of the learning process. As a result, predictors that might be overlooked with other methods can contribute to the prediction [31]. Squared prediction errors were estimated for each fold using the leave-one-out cross validation method [39] and then the root mean of the squared prediction error (RMSE) was calculated. In addition, we used a ten-fold cross validation test for comparing two models [40]. For this test, the original data was divided into ten subsets with equal numbers of patients in each subset; one subset was reserved as test data (to calculate prediction errors), and models were generated using the remaining nine ‘training data’ subsets. This process was repeated ten times so that each of the ten subsets was used once as test data. The above procedure was repeated ten times in total so that the accuracy of each model was estimated 100 times. The differences in prediction errors between the two models were compared; Z-values with ten degrees of freedom were used to obtain p-values.

For comparison with the Random Forest method, a series of linear models were generated. VRQoLscores were regressed against a single predictor variable: (i) IVF MD; (ii) VA of better-eye; (iii) VA of worse-eye; and against multiple predictor variables: (iv) IVF TDs, and VAs of better- and worse-eyes (multiple linear regression).

The impact of reduced VA, and diminished VF sensitivity in different regions of the IVF, on each VRQoLtask was determined using the Random Forest ‘Variable Importance’ measure; this was calculated by randomly permuting a variable at each decision tree and measuring whether the squared errors decreased [30]. The rank of importance of IVF test points were identified for each VRQoL task and overall VRQoL.

All statistical analyses were carried out using the statistical programming language R (ver. 2.15.0, The R Foundation for Statistical Computing, Vienna, Austria) and the ‘randomForest’ package (ver. 4.6–6) [41].

## Results

### Accuracy of Prediction of Vision-related Quality of Life

The RMSE associated with each model for each VRQoL task and overall VRQoL score are depicted in **Table 3**. Linear model prediction errors were smaller for the worse-eye VA compared with the better-eye VA, and significantly smaller for walking and going out tasks, and total VRQoL score (p-values were 0.33, 0.009, 0.02, 0.30, 0.07, for letters and sentences, walking, going out, dining, and total VRQoL, respectively). The RMSEs associated with the Random Forest method were significantly smaller than those by any other model for all tasks.

**Table 3:** RMSE for each VRQoL task and overall VRQoL score.

QoVL Task	VA (worse eye)	VA (better eye)	MD	MR	Random Forest
‘Letters’ and ‘Sentences’	0.98**	1.03**	0.94**	1.38**	0.84
‘Walking’	0.38**	0.43**	0.34**	0.49**	0.30
‘Going out’	0.40**	0.46**	0.42**	0.54**	0.32
‘Dining’	0.37*	0.40**	0.38*	0.52**	0.33
Overall	2.49**	2.77**	2.42**	3.38**	1.97

Each value is calculated as the absolute difference between predicted VRQoL score and the actual VRQoL score in the testing dataset in the leave-one-out cross validation. RMSE: root mean of the squared prediction error, VRQoL: vision-related quality of life, VA (worse-eye): visual acuity of the eye with worse mean deviation (MD), VA (better-eye): visual acuity of the eye with better MD, MR: Multiple regression with VA (better-eye), VA (worse-eye) and MD of IVF (Integrated Visual Field) (**: p<0.05, *: p<0.01, in comparison with Random Forest, ten fold cross validation).

### Areas of the Visual Field Important for Vision-related Quality of Life

**Figure 2:**
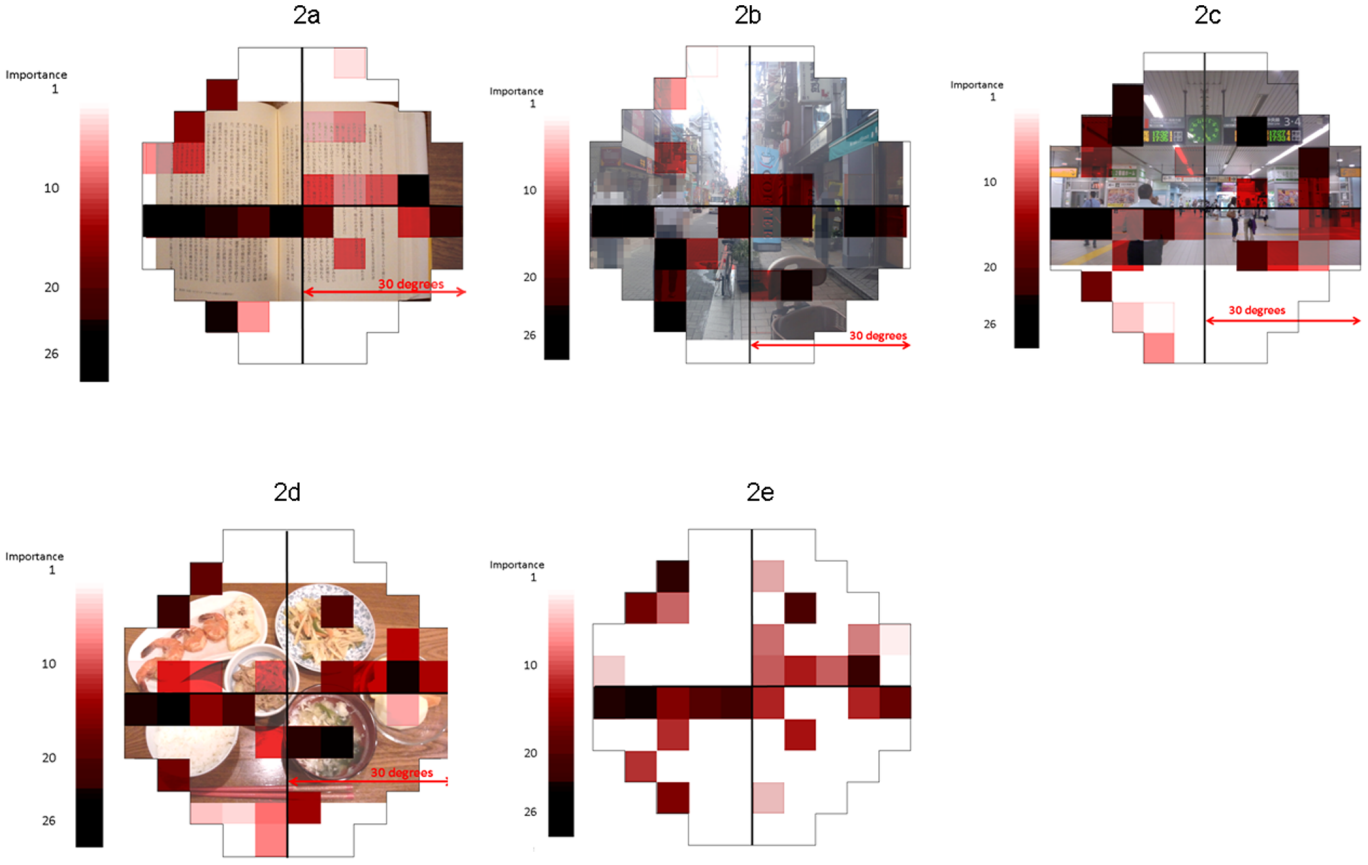
Rank importance, where impairment at a point has a significant association with decreased vision-related quality of life. The 26 important integrated visual field (IVF) test locations for each VRQoL task and overall VRQoL. IVF test points were superimposed onto an illustrative photograph corresponding to each task. The intensity of red increases according to the level of importance of each IVF test point. 1a: letters and sentences (viewing distance of 30 cm), 1b: walking (viewing distance of 5 m to the coffee shop flag, as viewed from the right hand side pavement, which is the walking direction in Japan), 1c: going out (viewing distance of 5 m to the information board), 1d: dining (viewing distance of 40 cm), 1e: total. Figure 2b has been edited to ensure anonymity of the people in it (the faces have been blurred) since written informed consent was not given.

Worse-eye VA was the most important variable in all VRQoL tasks and for general VRQoL (**Table S1**). Better-eye VA was the third most important factor for all VRQoL tasks except walking (**Table S1**). IVF location 40 (see **Figure 1**) was the second most important variable for all tasks and general VRQoL (**Table S1**). Important VF test points varied, according to the task. The 26 most important VF test points (approximately one-third of all 76 points in the total VF) for each task are illustrated in **Figure 2**. To visualize the correspondence between the identified IVF test locations and real life vision, IVF maps were superimposed onto photographs; distance to target was: 30 cm for the book as shown in **Figure 2a**, 5 m to the coffee shop flag as shown in **Figure 2b**, 5 m for the large information board shown in **Figure 2c**, and 40 cm for the plate of food shown in **Figure 2d**.

As suggested in **Figures 2a and 2b** and **Table S1 (online supplemental table)**, for the combined task of letters and sentences, and for the task of walking, important VF test points were concentrated along the horizontal meridian. In addition, test points in peripheral superior (10th, 11th, 13th and 20th important points) and inferior areas (5th and 21st important points) in the left hemifield were important for the letters and sentences task (**Figure 2a** and **Table S1**). For the task of walking, many test points ranked with high importance located in the peripheral, mid-peripheral and para-central inferior region (3rd, 4th, 6th, 9th, 13th and 14th important points) as well as test points along the horizontal meridian(**Figure 2b** and **Table S1**). For the task of going out, test points just beneath the horizontal meridian in the left and right hemifields and points in the peripheral superior region (2nd, 4th, 5th, 6th, 9th and 15th important points) were key points (**Figure 2c** and **Table S1**). For dining, important test points were scattered across the IVF, in addition to many test points along the horizontal meridian (**Figure 2d** and **Table S1**). The distribution of IVF test points important for overall VRQoL score was similar to that seen for the letters and sentences combined task (**Figure 2e** **)**. Central test points within 5° of the fixation tended to be chosen as heavily important for the task of letters and sentences (4th, 9th and 14th important points) and walking (8th, 11th and 17th important points), but not for the task of going out and dining (only 18th and 20th important points, respectively) (**Figure 2** and **Table S1**).

## Discussion

In the current study, glaucoma patients’ VRQoL was correlated with their IVF sensitivity and VA, and the Random Forest method yielded small prediction errors, compared to linear modeling approaches. We also identified the test points of the IVF that are most important for different daily tasks.

The linear model of worse-eye VA predicted VRQoL more accurately than the comparable models using better-eye VA or MD. Whether VA of better eye, or VA of worse eye, has a stronger influence on VRQoL remains controversial [7], [9], [11], [15], [42]. These previous reports investigated the influence of each index independently, however there is one study, that did analyze the VA of worse and better eyes simultaneously using multiple linear regression model and it has concluded that the VA of worse eye is more important for VRQoL [3]. Multiple linear regression does not consider the influence of correlated predictor variables, and assumes independence; if this assumption is not valid, the importance of each variable is not accurately calculated. [43] For example, if two variables are closely related, the true influence of one of them will be masked by the other. Our results suggest that worse-eye VA tended to have a greater impact on VRQoL than better-eye VA; however, our results cannot be directly compared to previous reports because, in the current study, better- and worse-eye VA were defined using MD values, not VA directly.

The distribution of important test points for the total VRQoL score (**Figure 2e** and **Table S1**) became similar to that for combined letter and sentence task (**Figure 2a** and **Table S1**). One of the possible reasons for this similarity can be attributed to the large number of questionnaires in this task (eight in 30). In addition, difficulty with near vision tasks, such as reading, is a frequent complaint from visually-impaired persons [44], including glaucoma patients; moreover it was found to be the most important priority in glaucoma patients [45], [46]. Sumi et al. reported that retinal sensitivity in the inferior hemifield within 5° of the fixation, and VA of better eye, are most closely correlated to a dissatisfaction regarding reading [15]. Our results also suggest that this area is important, however, other important test points were also identified in the peripheral region. Indeed there is a report which suggested even further peripheral region is important for reading; reading ability starts to deteriorate when VF damage reaches to 50-degrees (with the II-4-e target in Goldmann perimetry)in patients with retinitis pigmentosa [47]. Our results suggest that it is very important to maintain VF sensitivity along the horizontal meridian, in particular in the left inferior hemifield, for the task of reading. In addition, test points in the inferior and superior peripheral regions in the left hemifield are also important (**Figure 2a** and **Table S1**). This finding is particularly interesting when we consider that Japanese literature is written in two directions: horizontally and vertically. When written horizontally, the first line begins at the top of the page, and each line is read left to right (exactly the same as English). When written vertically, the first line begins on the right hand side of the page, and is read top to bottom, and succeeding lines, to the left hand side, are then read in this manner. Thus, inferior and superior peripheral regions may play an important role in searching the next area of text when reading. In an eye movement tracking study, researchers found that in subjects reading ‘Manga’ (Japanese comics), which is written vertically, the eye fixation point moves rapidly from the bottom right hand side of the page to the top left hand side of the page, and restricting the viewing window to exclude these two areas resulted in reduced reading speeds [47], [48]. Furthermore, these peripheral regions play an important role in searching when reading a large page, such as a newspaper. Also it is well known people carry out “search performance” using peripheral vision when they find out a place to focus [49]. Thus, it may be beneficial to be able to see the outer shape of a book or article before concentrating on the place to read, which may explain why the test points in the superior and inferior peripheral areas and left and right peripheral points along the horizontal meridian. Although the questionnaire used in the current study is identical to the one in Sumi et al. [15], the findings obtained were quite different with the most important VF test points distributed more widely across the IVF in our results. We propose that this may be attributed to our application of the Random Forest method in the current study, which can interpret IVF sensitivity and VA simultaneously, while not biasing the results.

Similarly to the combined task of letters and sentences, the IVF region along the horizontal meridian was also important for the task of walking. The test points in the central area within 5° of fixation were given high importance, however, interestingly, we found that there were many test points with higher importance rank in the peripheral and mid-peripheral areas in the inferior hemifield (**Figure 2b**) rather than the test points in the central region, in contrary to Sumi’s report which suggested the central area is most important for this task. [15]. This may be because the visual function in the central area is compensated by VA, and so the key IVF locations are instead scattered in the inferior peripheral region. This finding is also in agreement with a previous study which suggested the importance of this area for postural stability [17]. Many reports have suggested that people with glaucoma walk more slowly than people without ocular disease [50], [51]. Our results suggest that glaucoma patients with VF deterioration in the inferior peripheral area should be advised about walking problems. This is clinically important, because it has been reported that the risk of hip fractures is increased in glaucoma patients [52].

The task of ‘going out’ is unique to the Sumi questionnaire. This task mainly addresses the ability to find targets when travelling, such as information boards or tube maps at a train station, or a traffic signal when crossing a road. In metropolitan areas, such as Tokyo, it is very common to use public transportation rather than a private motor car, hence we would expect this particular task has a significant implication upon a person’s quality of life. These targets usually need to be viewed from a long distance and are often located in a high position. With this in mind, it is very interesting that many test points in the upper peripheral and mid-peripheral IVF regions were selected as highly important, in addition to IVF points along the horizontal meridian and test points in the central area within 5° of the fixation were not selected with heavy importance which disagree with what reported by Sumi et al [15].

For the task of dining, IVF points along the horizontal meridian (left and right peripheral areas) and test points in the paracentral area in the inferior right hemifield were given high importance, as well as other peripheral areas. As shown in the **Figure 2d**, these test points overlap to rice bowl (1st, 4th, 7th, 10th, 12th, 17th and 19th important points), miso soup (2nd, 5th and 16th important points), main dish (7th and 19th), side dishes (6th, 9th, 11th, 19th, and 8th, 11th, 16th, and 3rd, 14th, 15th, 16th, 23rd important points) and chop sticks (13th, 21st, 24th and 25th important points). Interestingly this is not in agreement with Sumi et al. [15], where test points in the central area were selected as most important. In traditional Japanese dining, the rice bowl is held in the left hand. During eating, the rice bowl is repeatedly picked up and put down in one’s left hand. This may explain why the selected IVF regions in **Figure 2d** are important for dining. In Western countries, we would expect that different IVF regions are more important, most likely the central area.

The VRQoL score observed for each different task is independent yet test point 40 was given the second highest importance for all of the tasks and overall VRQoL score as shown in Figure 2, and neighboring points were also given relatively high ranks. This suggests that test points just beneath the horizontal meridian in the left hemifield are important for many tasks in daily life and careful attention should be made when clinicians see patients with VF damage in this area.

Several classification tools, such as support vector machines, could also have been used for the purpose of this research. Previous reports support the use of the Random Forest method [53]–[55], however, future work is necessary to investigate which method is most appropriate to analyze the relationship between visual function and VRQoL. A limitation of the current study is that the Sumi Questionnaire does not include questions about driving. Outside of metropolitan areas like Tokyo, driving is an important feature of daily life, hence this task should be addressed in a future study. Another limitation in the current study is that we have merged the tasks of ‘letters’ and ‘words’ since the questions in these sections are very be similar in content, however, this has not been validated so this should be investigated in future. An important caveat of this study is that the most important VF test locations for different VRQoL tasks may be different in advanced glaucoma patients. A further study should be carried out in a sample of advanced glaucoma patients with a more constricted VF and damage near fixation, because more central areas (near fixation) of the VF could be more important in these patients. Conversely, the same study carried out in an early stage glaucomatous population would also be beneficial, because the deterioration of VRQoL in these patients may be attributed to different location areas of the VF.

### Conclusion

In conclusion, we have analyzed the relationship between VF sensitivity and VA, and VRQoL. The Random Forest method takes into account the interrelationships of the data and gives an accurate prediction of VRQoL. Most importantly, the model enables clinicians to better understand patients’ VRQoL based on standard clinical measurements. Furthermore, VF test points critical for different daily tasks and VRQoL were identified. These results will help clinicians to concentrate on these regions when managing patients, and offer appropriate advice if these areas are damaged. These findings should be considered when welfare policy is decided by public administration, such as positioning information boards in a train station, and providing walking guides for the visually disabled. Moreover, this prediction system could be used to estimate VRQoL for the different tasks of daily life for a given patient, using only conventional VA and VF measurements.

## Supporting Information

Table S1 **The rank of the importance of integrated visual field (IVF) test points, visual acuities of better and worse eyes, gender and age.** The numbers represent the IVF test point locations (see Figure 1). VA (worse-eye): visual acuity of the eye with worse mean deviation (MD) and VA (better-eye): visual acuity of the eye with better MD.
(XLS)
