# Correction: Identifying Areas of the Visual Field Important for Quality of Life in Patients with Glaucoma

**DOI:** 10.1371/journal.pone.0144212

**Published:** 2015-11-30

**Authors:** Hiroshi Murata, Hiroyo Hirasawa, Yuka Aoyama, Kenji Sugisaki, Makoto Araie, Chihiro Mayama, Makoto Aihara, Ryo Asaoka

There is an error in Fig 2. The heatmap intensity reference bars are incorrectly positioned. Please view the correct Fig 2 here.

**Fig 2:**
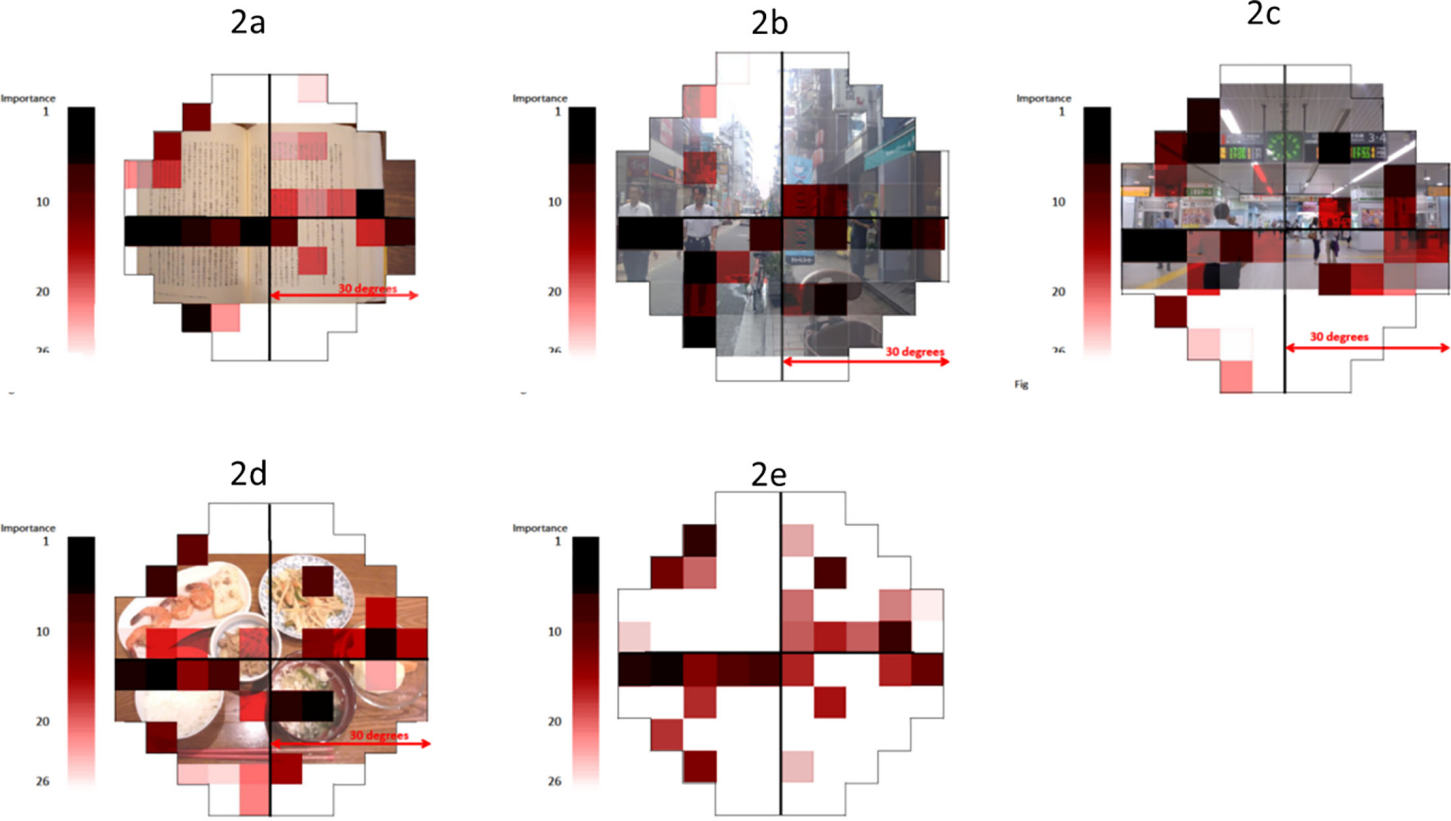
Rank importance, where impairment at a point has a significant association with decreased vision-related quality of life.

The 26 important integrated visual field (IVF) test locations for each VRQoL task and overall VRQoL. IVF test points were superimposed onto an illustrative photograph corresponding to each task. The intensity of red increases according to the level of importance of each IVF test point. 2a: letters and sentences (viewing distance of 30 cm), 2b: walking (viewing distance of 5 m to the coffee shop flag, as viewed from the right hand side pavement, which is the walking direction in Japan), 2c: going out (viewing distance of 5 m to the information board), 2d: dining (viewing distance of 40 cm), 2e: total. Fig 2B has been edited to ensure anonymity of the people in it (the faces have been blurred) since written informed consent was not given.
